# Impact of different perioperative dexmedetomidine administration regimens on postoperative sleep quality in gastrointestinal tumor resection: a randomized controlled trial

**DOI:** 10.20452/wiitm.2025.17966

**Published:** 2025-07-04

**Authors:** Chengying Ji, Xiaodong Su, Chaohui Gao, Qijing Liu, Ying Liu, Qian Fu, Boxiong Gao, Jiayi Xie, Bokang Yang, Jinxiang Xie, Huping Song, Yatao Liu

**Affiliations:** The First School of Clinical Medicine, Lanzhou University, Lanzhou, Gansu, China; Department of Anesthesiology and Surgery, First Hospital of Lanzhou University, Lanzhou, Gansu, China

**Keywords:** analgesic pumps, dexmedetomidine, gastrointestinal tumor surgery, postoperative sleep quality, randomized controlled trial

## Abstract

**INTRODUCTION:**

Perioperative sleep disorders constitute a recognized risk factor for multiple postoperative complications. Although dexmedetomidine (DEX) has been clinically employed to enhance perioperative sleep quality, its optimal administration protocol for postoperative sleep improvement remains undetermined.

**AIM:**

The aim of this study was to comparatively evaluate the therapeutic effect of distinct perioperative DEX administration strategies on postoperative sleep quality in patients undergoing elective gastrointestinal tumor resection via laparoscopy.

**MATERIALS AND METHODS:**

A total of 48 patients undergoing laparoscopic gastrointestinal resection between September 2024 and January 2025 were enrolled and randomly allocated to the intraoperative continuous DEX infusion group (group I; n = 24) and the group with DEX added to postoperative intravenous analgesia (group P; n = 24) using a double-blind method. Sleep quality was assessed using the Numerical Rating Scale during the first 3 postoperative days. A comparative analysis of intergroup differences in postoperative sleep quality was performed.

**RESULTS:**

Out of the 48 randomized participants, 47 were included in the analysis, as 1 patient from group P withdrew informed consent postoperatively. Baseline data were balanced between the 2 groups. In comparison with group I, on postoperative day 1, group P exhibited considerably higher sleep quality scores (*P* = 0.045), lower blood glucose levels at skin suture completion (*P* <⁠0.001), higher intraoperative norepinephrine doses (*P* <⁠0.001), and reduced intraoperative blood loss (*P* = 0.03). Multivariable linear regression identified group assignment (*P* = 0.03) and sex (*P* = 0.02) as significant predictors of sleep quality on postoperative day 1.

**CONCLUSIONS:**

As compared with intraoperative continuous DEX infusion, addition of DEX to postoperative analgesia in laparoscopic gastrointestinal tumor surgery has better outcomes with regard to sleep quality on postoperative day 1. These findings suggest potential advantages of postoperative DEX administration in perioperative management.

## INTRODUCTION

Studies have shown that up to 60% of patients undergoing surgery experience postoperative sleep disorders, with the rapid eye movement stage tending to decrease or disappear completely during the first night after surgery, while exhibiting rebound intensity and duration over subsequent nights.^1^^,^^2^^,^^3^ Postoperative slow-wave sleep is significantly reduced, and these sleep disorders and architectural disruptions strongly correlate with multisystem dysfunction, including neurological complications, such as postoperative delirium and cognitive dysfunction. Improving postoperative sleep quality in surgical patients can mitigate or even prevent complications triggered by sleep disturbances, thereby enhancing prognosis and quality of life.^4^^,^^5^^,^^6^ In the central nervous system (CNS), catecholaminergic neurons in the locus coeruleus (LC)—the primary source of norepinephrine (NE)—exhibit extensive cortical synaptic projections, while adrenergic neurons in the medullary reticular formation (expressing β1 / β2 receptors) drive CNS-wide alertness via α1 receptor activation, contrasting with presynaptic inhibitory / sedative effects of α2 receptors.^7^

α2 adrenergic receptor agonists improve sleep quality by activating α2 receptors to reduce depolarization rates of LC neurons in the CNS, thereby decreasing arousal-promoting adrenergic inputs to the cerebral cortex, basal forebrain, thalamus, and hypothalamic preoptic area. This reduced signaling activates sleep-active neurons that inhibit brainstem arousal nuclei via γ-aminobutyric acid and galanin secretion, while directly targeting noradrenergic neurons in the thalamus and basal forebrain to promote sleep.^8^

Dexmedetomidine (DEX) mimics natural sleep pathways through central α2 adrenergic receptor activation, directly increasing nonrapid eye movement stage N3 (deep sleep) duration,with its postoperative analgesic pump–mediated sedation extending into recovery to reduce awakenings and enhance subjective sleep perception.^9^

Current studies on using DEX to improve postoperative sleep quality primarily focus on 2 administration methods: intraoperative continuous intravenous infusion or postoperative addition to a patient-controlled intravenous analgesia (PCIA) pump, yet reported sleep quality improvements remain inconsistent across trials.^10^^,^^11^^,^^12^^,^^13^**^,^**^13^**^,^**^14^ The mechanism by which each of these 2 drug regimens improves sleep quality is unclear. The absence of direct comparisons between these DEX application modes prompted our randomized trial design to identify the optimal perioperative administration strategy, while exploring underlying mechanisms.

## AIM

The study aimed to compare the therapeutic impact of distinct perioperative DEX administration strategies on postoperative sleep quality in patients undergoing elective gastrointestinal tumor resection via laparoscopy.

## MATERIALS AND METHODS

### Trial design

This randomized controlled trial compared continuous intraoperative DEX infusion (group I) with postoperative addition of DEX to intravenous analgesia (group P) to assess their effects on early postoperative sleep quality in gastrointestinal tumor patients. A total of 48 participants were randomized into 2 groups at a 1:1 ratio.

### Participants

The study included patients aged 18–70 years who underwent elective laparoscopic gastrointestinal tumor resection at the First Hospital of Lanzhou University between September 2024 and January 2025 and agreed to the use of a PCIA pump.

Exclusion criteria comprised: 1) a history of sleep disorders; 2) preoperative use of sleep-promoting medications 1 month prior to surgery or a Pittsburgh sleep quality index (PSQI) score higher than or equal to 6^15^; 3) preoperative long-term use of opioids, sedatives, antidepressants, or anxiolytics; 4) a history of depression, schizophrenia, epilepsy, or Parkinson disease; 5) incapability of communication (coma, severe dementia, or language impairment); 6) sick sinus syndrome, sinus bradycardia (heart rate <⁠50 bpm), or second-degree or greater atrioventricular block without a pacemaker; 7) Child–Pugh class C liver function; 8) severe renal dysfunction requiring dialysis; 9) myasthenia gravis; 10) allergy to opioid analgesics or DEX; 11) a history of alcohol abuse or prolonged, heavy drinking with a consumption of more than 3 units of alcohol per day; 12) excessive consumption of tea (≥500 ml per day), coffee (≥400 ml per day), or energy drinks (≥250 ml per day); and 13) failure to sign the informed consent form.

### Interventions

Preoperative preparation involved visits conducted 24 hours prior to surgery, which included medical record review, trial purpose explanation, risk disclosure, and informed consent acquisition from patients / legal guardians, followed by baseline data collection (sex, age, height, weight). On the day of surgery, preoperative randomization determined trial drug preparation, with subsequent peripheral venous access establishment, radial artery cannulation under local anesthesia for invasive blood pressure monitoring, and concurrent electrocardiography / oxygen saturation / anesthesia depth monitoring.

Group I received intravenous DEX (1 μg/kg/10 min; Jiangsu Hengrui Medicine Co., Ltd, Lianyungang Jiangsu, China) alongside propofol (2 mg/kg), and sufentanil (0.5 μg/kg), with rocuronium (0.6 mg/kg) administered after the anesthesia index reduction to a value below or equal to 60. Group P received an equivalent amount of saline instead of DEX. Tracheal intubation commenced 3 minutes after rocuronium administration under volume-controlled ventilation (tidal volume, 8–10 ml/kg; heart rate, 12 bpm; end-tidal carbon dioxide, 35–45 mm Hg).

To maintain the anesthesia, group I received continuous DEX (0.4 μg/kg/h), propofol (4–12 mg/kg/h), and remifentanil (3–120 μg/kg/h), while group P was given saline placebo. Both groups received rocuronium (0.3–0.6 mg/kg/h) and maintained anesthesia index values at 40–55 via the attending anesthesiologist titration. Intraoperative thermoregulation (nasopharyngeal temperature, 36–37 °C) and hemodynamic control (ca. 20% baseline heart rate) were enforced, with vasoactive agents (NE / urapidil / atropine) administered as needed. DEX infusion ceased 30 minutes before suture completion, with intraoperative blood glucose testing and ondansetron (4 mg) and flurbiprofen axetil (50 mg) administered at wound closure. All anesthetic infusions terminated after suturing.

Postoperative analgesia involved PCIA pumps delivering 72-hour analgesia. Group P received sufentanil (0.04 μg/kg/h),^14^ DEX (0.04 μg/kg/h), flurbiprofen axetil (0.04 mg/kg/h), and ondansetron (0.1 mg/kg/72 h) in 150 ml of saline. Group I received an identical regimen except for DEX. PCIA parameters included a 2-ml bolus, 2 ml/h basal rate, a 2-ml demand dose, and a 15-min lockout. Pump labeling (patient name / hospitalization number) ensured blinding. Rescue analgesia (intravenous flurbiprofen axetil 50 mg) was administered for visual analog scale (VAS) scores above 4, unresponsive to PCIA.

Data were collected using validated tools at bedside assessments (subjective sleep quality, resting pain scores, delirium screening) which were conducted daily from 8:00 to 9:00 AM during the first 3 postoperative days. Sleep quality was evaluated using the Numerical Rating Scale (NRS) of 0–10, where 0 meant the worst sleep quality, and 10 corresponded to the best sleep quality.^16^ Pain was assessed with the VAS of 0–10 points, where 0 equaled no pain and 10 meant the worst pain.^17^ Delirium was evaluated via the 3-min diagnostic confusion assessment method (3D-CAM),^18^ whereas adverse events were recorded during structured interviews with the patients, families, and hospital ward staff. Blood glucose at the end of surgery was measured from the venous blood collected during wound closure for biochemical testing. Inflammatory markers included white blood cell count (WBC), C-reactive protein (CRP) and blood glucose levels on postoperative day 1, analyzed based on venous blood samples collected at 7:00 AM, using routine hematological and biochemical assays.

### Outcomes

The primary outcome was the subjective sleep quality on the first postoperative day.

Secondary outcomes involved subjective sleep quality on the second and third postoperative days, resting pain levels during the first 3 postoperative days, blood glucose at the end of surgery, and inflammatory markers (WBC, CRP) on postoperative day 1. Incidence of delirium (assessed via the 3D-CAM scale) and postoperative adverse events (hypotension, bradycardia, nausea, and vomiting) were evaluated during the first 3 postoperative days.

### Sample size

Based on preliminary data showing mean (SD) intergroup difference of 3.3 (3.61) in sleep quality NRS scores, the sample size was calculated according to Chow et al^19^ as follows: α = 0.05, power [1–β] = 0.8, group ratio 1:1, yielding 19 patients per group. Accounting for a 20% attrition rate, 24 patients were enrolled in each group.

### Randomization

A computer-generated randomization sequence was used to allocate patients at a 1:1 ratio to group I or group P. The randomization results were sealed in sequentially numbered opaque envelopes by nurse anesthetists uninvolved in the study. A blinded researcher screened and enrolled eligible patients on the day of surgery according to the envelope order, while the nurse anesthetist opened the assigned envelope to prepare study medications. These nurses remained excluded from intraoperative procedures, postoperative assessments, and data collection. The patients, anesthetists, and outcome assessors remained blinded until the study completion. All study medications were administered via identical 150-ml colorless analgesic-pump reservoir bags, using devices of the same brand to eliminate intergroup visual differences.

**Statistical analysis** This study used a per-protocol sets analysis. Normally distributed data are presented as mean (SD) and were analyzed using the independent *t* tests, while non-normally distributed continuous and ordinal data are expressed as medians (interquartile range [IQR]) and were compared using the Wilcoxon rank-sum test. Categorical variables are reported as numbers (percentages), with intergroup differences assessed using the χ^2^ or the Fisher exact tests. A *P *value below 0.05 was deemed significant. Variables were initially screened by univariable linear regression. The ones with a *P* value below 0.05, along with other clinically relevant variables, were included in the multivariable linear regression analyses. Prespecified subgroup analyses (stratified by sex, chemotherapy status, stoma status, and year of surgery) were used to explore potential heterogeneity in the effects of sleep interventions. Given sample size limitations, these analyses were exploratory and not used for formal hypothesis testing. All analyses were conducted using SPSS Statistics software, version 27.0 (IBM, Armonk, New York, United States).

### Ethics

This study received ethical approval from the Institutional Review Board of the First Clinical Medical College, Lanzhou University (LDYYLL2024-344) and all patients included in the study signed an informed consent form. This study was prospectively registered with the Chinese Clinical Trial Registry (ChiCTR2400089184; https://www.chictr.org.cn).

## RESULTS

### Patient inclusion and demographic characteristics

**FIGURE 1:**
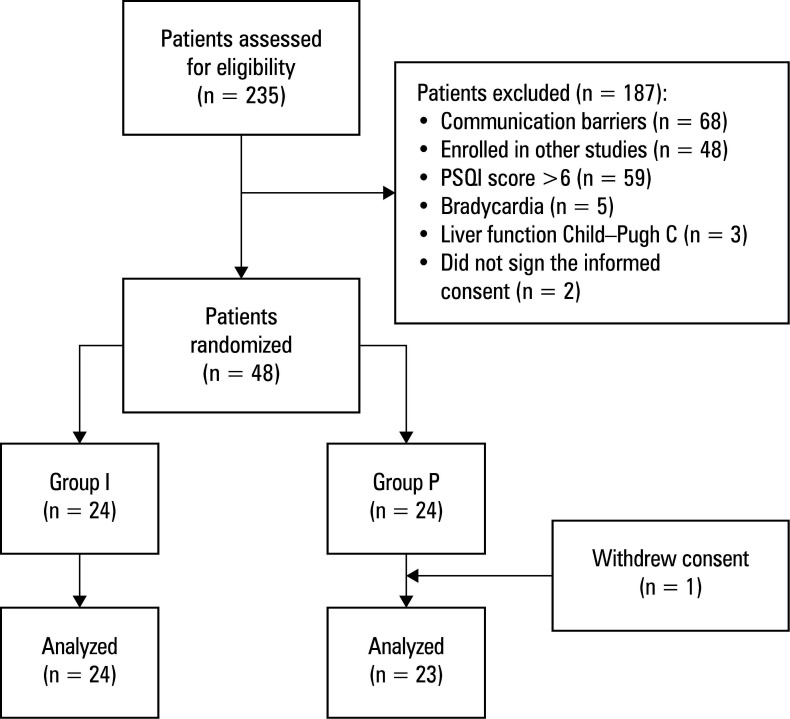
Flow chart of patient selection and group allocation

Patient screening process was conducted from September 1, 2024 to January 17, 2025. Out of 235 screened patients, 48 met the eligibility criteria and were randomized into 2 groups. One patient from group P withdrew informed consent postoperatively, resulting in 47 patients being included in the final analysis (FIGURE 1).

Abrreviations: PSQI, Pittsburgh sleep quality index

**TABLE 1:** Baseline characteristics of the groups

Variable		Group I (n = 24)	Group P (n = 23)	P value
ASA classification	I	19 (79.17)	20 (86.96)	0.75
	II	5 (20.83)	3 (13.04)	
Sex	Men	16 (66.67)	18 (78.26)	0.37
	Women	8 (33.33)	5 (21.74)	
Age, y		59.5 (54.25–64)	58 (48–62)	0.42
BMI, kg/m², mean (SD)		26.99 (2.98)	26.04 (2.12)	0.21
PSQI scores		2 (2–3.75)	2 (2–5)	0.67
Surgical location	Stomach	13 (54.17)	11 (47.83)	0.77
	Colorectal area	11 (45.83)	12 (52.17)	
Preoperative medical history				
Smoking status		0	3 (13.04)	0.11
Diabetes		2 (8.33)	0	0.49
Hypertension		5 (20.83)	2 (8.7)	0.45
Coronary heart disease		1 (4.17)	1 (4.35)	>0.99
Chemotherapy		11 (45.83)	10 (43.48)	0.87
Laboratory parameters				
Blood glucose, mmol/l		5.8 (4.9–6.1)	5.2 (5–5.5)	0.1
White blood cell count, × 10⁹/l		4.67 (3.67–6.16)	5.51 (4.09–5.91)	0.23
Hemoglobin, × 10⁹/l		132.5 (111–147.5)	129.09 (101–151)	0.92
Alanine aminotransferase, U/l		21 (17–24.75)	20 (15–30)	0.86
Serum creatinine, μmol/l, mean (SD)		68.37 (13.83)	69.44 (13.76)	0.79
Serum albumin, g/l, mean (SD)		43.15 (3.36)	42.54 (3.41)	0.54

Data are presented as numbers (percentages) or median (interquartile range) unless indicated otherwise.

Abbreviations: ASA, American Society of Anesthesiologists; BMI, body mass index; others, see FIGURE 1

No differences were observed between the groups in the American Society of Anesthesiologists classification involving sex, age, body mass index, PSQI score, smoking status, comorbidities (diabetes mellitus, hypertension, coronary artery disease), preoperative chemotherapy, WBC count, and the levels of hemoglobin, alanine aminotransferase, creatinine, serum albumin, or glucose TABLE 1.

### Intraoperative characteristics

Both groups exhibited comparable surgical / anesthesia durations, intraoperative medication dosages (propofol, rocuronium, sufentanil, and remifentanil), crystalloid infusion volumes, urine output, fistula status, and surgery time. However, group P required significantly higher intraoperative NE doses than group I (median [IQR], 650.5 [280−292] vs 188 [8−310] µg), whereas group I had significantly greater median (IQR) intraoperative blood loss, as compared with group P (200 [100–387.5] vs 100 [80−200] ml; TABLE 2).

### Outcomes and estimation

**FIGURE 2:**
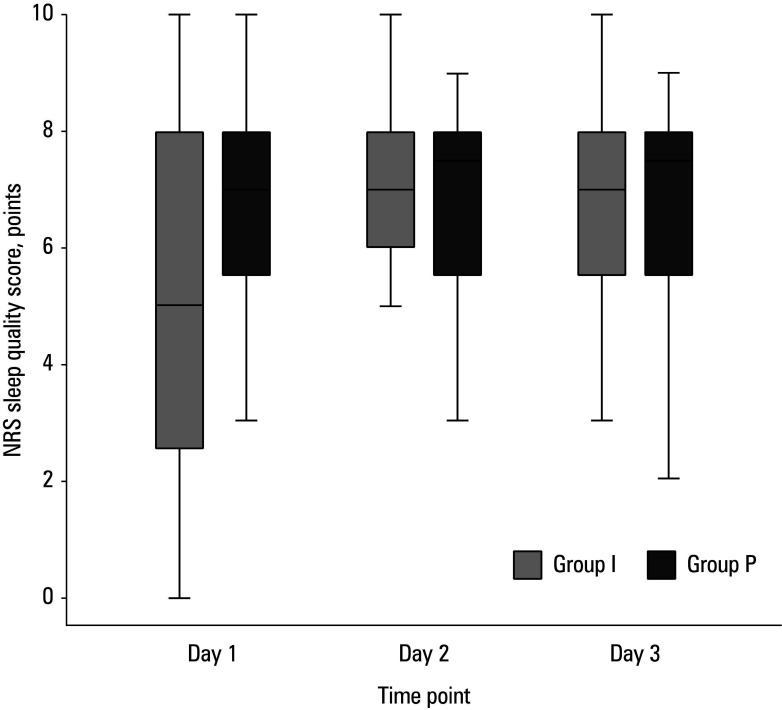
Sleep quality on the first 3 postoperative days. Whiskers indicate the minimal and maximal values, boxes indicate the interquartile range, and the line in the middle of each box represents the median.

Abbreviations: NRS, Numerical Rating Scale

On postoperative day 1, the patients in group P exhibited markedly higher median (IQR) sleep quality NRS scores than group I (7 [5−8] vs 5 [2.3–8]; TABLE 3), whereas no intergroup differences were observed on postoperative days 2 and 3 (FIGURE 2). Pain scores assessed from postoperative days 1 to 3 showed no differences between the groups. At surgical conclusion, group P demonstrated lower mean (SD) arterial blood glucose levels than group I (6.01 [0.89] vs 7.19 [1.24] mmol/l; *P *<⁠0.001); however, no differences in CRP, WBC count, or blood glucose values were observed on postoperative day 1 (TABLE 4).

### Safety

No intergroup differences were observed in the incidence of hypotension, bradycardia, postoperative delirium, nausea, or vomiting, or the requirement for postoperative emergency analgesia during the first 3 postoperative days (TABLE 5).

### Univariable and multivariable analyses

Although the sleep quality analysis data on the first postoperative day resulted skewed, their residuals were tested to be consistent with the assumption of normal distribution, so linear regression was applied for statistical analysis.

In the univariable analysis, group assignment (group P vs group I, β = 1.74; 95% CI, 0.35–3.14) and female sex (women vs men, β = −2.2; 95% CI, −3.7 to −0.67) demonstrated significant effects on postoperative day 1 sleep quality, whereas age, remifentanil dosage, and postoperative day 1 pain scores were insignificant (TABLE 6).

Several previous studies have shown that age, opioid dosage, and postoperative pain level are key factors affecting postoperative sleep quality.^20^**^,^**^21^ Therefore, along with the 2 variables listed above, they were also included in the multivariable linear regression analysis. Multivariable regression confirmed that postoperative day 1 sleep quality was significantly associated with group assignment (β = 1.61; 95% CI, 0.22–3 and sex (β = −1.94; 95% CI, −3.52 to −0.35), whereas age, remifentanil dosage, and postoperative day 1 pain scores remained insignificant (TABLE 7).

**TABLE 2:** Intraoperative data

Variable		Group I (n = 24)	Group P (n = 23)	P value
Duration of surgery, min, mean (SD)		247.67 (91.19)	236.39 (91.94)	0.68
Duration of anesthesia, min, mean (SD)		269.08 (94.19)	255.3 (91.33)	0.61
Intraoperative drug dosage	Propofol, mg, mean (SD)	973.01 (395.55)	1145.19 (400.14)	0.15
	Sufentanil, μg	40 (35–43)	40 (35–50)	0.74
	Remifentanil, μg	3027 (2172.5–4305)	3646.7 (2850–3915)	0.38
	Rocuronium, mg	90 (65.25–115)	98 (80–112)	0.48
	Norepinephrine, μg	188 (81–310)	650.5 (280–892)	1
	Crystalline liquid, ml, mean (SD)	1864.58 (707.18)	1778.26 (585.59)	0.65
Blood loss, ml		200 (100–387.5)	100 (80–200)	0.03
Urine output, ml		400 (250–900)	500 (250–850)	0.93
Fistula		19 (79.17)	20 (86.96)	0.75
Surgery time	Morningª	20 (83.33)	16 (69.57)	0.44
	Afternoonᵇ	4 (16.67)	7 (30.43)	

Data are presented as numbers (percentages) or median (interquartile range) unless indicated otherwise.

a Surgery completed by 12:00 PM

b Surgery not completed by 12:00 PM

**TABLE 3:** Sleep quality according to the Numerical Rating Scale

Parameter	Group I (n = 24)	Group P (n = 23)	P value
Postoperative day 1	5 (2.3–8)	7 (5–8)	45
Postoperative day 2	7 (6–8)	7.5 (5.75–8)	0.92
Postoperative day 3	7 (5.5–8)	7.5 (5.75–8)	0.94

Data are presented as median (interquartile range).

**FIGURE 3:**
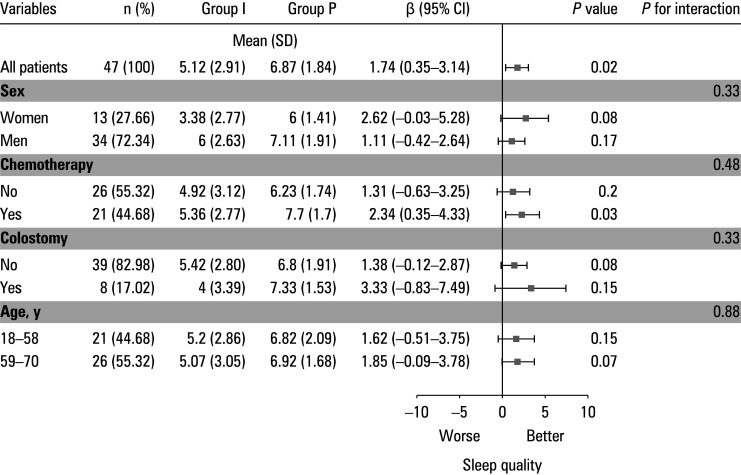
Subgroup analysis forest map

Subgroup analyses of postoperative day 1 sleep quality stratified by sex, chemotherapy status, stoma status, and age (median-based dichotomization) showed no differences between the subgroups and insignificant effect of heterogeneity across the subgroups (FIGURE 3).

## DISCUSSION

While intraoperative continuous DEX infusion and addition of DEX to postoperative intravenous analgesia are commonly employed in clinical studies to enhance postoperative sleep quality, no prior research has directly compared these 2 administration modalities—a gap addressed by this randomized trial evaluating their differential effects on sleep outcomes in gastrointestinal tumor surgery patients.

As a selective α2-adrenoceptor agonist, DEX improves sleep architecture while attenuating inflammatory responses, surgical stress, and postoperative pain intensity. By assessing these outcomes under distinct DEX regimens and analyzing multidimensional factors influencing sleep quality, our study provided mechanistic insights to optimize clinical drug regimens.

### Sleep

Addition of DEX to postoperative intravenous analgesia significantly improved sleep quality on postoperative day 1, as compared with intraoperative continuous DEX infusion (0.4 μg/kg/h) in patients undergoing gastrointestinal tumor surgery, while no intergroup differences were observed on postoperative days 2 and 3.

In clinical practice, intraoperative DEX is typically administered at an initial 1 μg/kg loading dose over 10–15 minutes, followed by continuous infusion (0.2–0.7 μg/kg/h). Lu et al^13^ demonstrated that intraoperative DEX at 0.2 μg/kg/h markedly enhanced NRS-based sleep quality on postoperative days 2 and 5 in comparison with saline controls, with shorter time to first postoperative flatus. Real-world evidence^10^ has indicated that low-dose intraoperative DEX (0.2–0.4 μg/kg/h) reduced severe sleep disorders on postoperative day 1 without affecting operative duration, 24-hour postoperative nausea / vomiting, bowel function, or analgesic requirements. Based on these findings, this study employed intraoperative DEX at a dose of 0.4 μg/kg/h.

A 2023 study by Wang et al^22^ demonstrated that a microdose of DEX (0.02 μg/kg/h) in analgesic pumps in hospital patients improved sleep architecture but showed no significant effect on sleep quality NRS scores during postoperative days 1–5. Sui et al^14^ found that postoperative combined analgesia with sufentanil and DEX (200 μg or 400 μg of DEX diluted in 300 ml, of saline infused at 4 ml/h) enhanced Athens Insomnia Scale scores over postoperative days 1–7 and accelerated gastrointestinal recovery in surgical patients, as compared with sufentanil alone. Another randomized trial^23^ showed that combined analgesia with morphine and DEX (2 μg/h) considerably improved NRS sleep quality scores on postoperative day 1 in comparison with morphine-only regimens. Based on these findings, we adopted a postoperative DEX infusion dose of 0.04 μg/kg/h.

### Stress

A meta-analysis^24^ demonstrated that continuous intraoperative DEX infusion reduced 24-hour postoperative blood glucose levels by attenuating surgical stress responses through modulation of the hypothalamic-pituitary-adrenal axis and sympathoadrenal medullary axis, whereas another study^25^ suggested DEX may elevate glucose levels via pancreatic β-cell inhibition of insulin secretion.^26^ In this trial, the patients receiving intraoperative DEX infusion exhibited significantly higher blood glucose levels at surgical conclusion than the group with DEX added to postoperative analgesia, though this difference became insignificant by postoperative day 1. These dual glycemic effects likely reflect dose- and timing-dependent pharmacodynamics, indicating that sustained intraoperative DEX administration carries perioperative hyperglycemia risks.

**TABLE 4:** Other outcomes

Outcome		Group I (n = 24)	Group P (n = 23)	P value
Pain, VAS score, points	Postoperative day 1	3 (2–5.8)	3 (1–5)	0.28
	Postoperative day 2	4.5 (4–5.25)	4.5 (4–5)	0.6
	Postoperative day 3	4.58 (3–4.58)	4.58 (3–5)	0.68
Blood glucose, mmol/l	Day of surgery	7.19 (1.24)	6.01 (0.89)	<0.001
	Postoperative day 1	6.1 (4.99, 7.56)	4.97 (4.36, 7.69)	0.12
White blood cell count, postoperative day 1, × 10⁹/l		10.97 (7.66–12.81)	10.35 (8.98–13.28)	0.5
C-reactive protein, postoperative day 1, mg/l		39.38 (19.72–44.33)	39.38 (25.83–51.72)	0.68

Data are presented as mean (SD) or median (interquartile range).

Abbreviations: VAS, visual analog scale; others, see TABLE 2

**TABLE 5:** Adverse events

Adverse event		Group I (n = 24)	Group P (n = 23)	P value
Intraoperative	Hypotension	0	0	>0.99
	Bradycardia	0	0	>0.99
Postoperative	Hypotension	0	0	>0.99
	Bradycardia	0	0	>0.99
	Delirium	1 (2.13)	1 (4.35)	0.49
	Nausea and vomiting	0	0	>0.99

Data are presented as numbers (percentages).

### Blood loss and vasoactive drugs

Significant intergroup differences were observed in intraoperative blood loss and NE requirements, with greater blood loss in group I and greater NE utilization in group P, findings potentially attributable to bidirectional blood pressure modulation of DEX.^27^ Li et al^28^ showed that DEX had a better clinical effect on improving perioperative hemodynamics in patients who underwent laparoscopic surgery. These results further indicate that intraoperative DEX infusion at 0.4 μg/kg/h provides superior hemodynamic stability, as compared with nonintraoperative DEX administration.

### Differences based on sex

Linear regression analysis demonstrated a negative association between female sex and postoperative day 1 sleep quality in comparison with male sex (β = –2.2; 95% CI, –3.74 to –0.67; *P* = 0.01), supporting the premise that female sex constitutes an independent risk factor for postoperative sleep disorders.**^29^**

### Pain

Postoperative pain, a key determinant of sleep quality,^5^ is mitigated by DEX through inhibition of nociceptive signaling pathways (eg, blockade of spinal NE release),^30^ thereby reducing pain-related sleep disruption. Wang et al^22^ demonstrated that there was no significant difference between postoperative DEX-assisted analgesia at 0.02 μg/kg/h and DEX-free regimens in relation to resting pain scores over postoperative days 1–5, whereas a higher DEX dose (4 μg/h) markedly improved resting pain at 12, 24, and 48 hours after surgery. In this study, the absence of intergroup differences in pain scores during postoperative days 1–3 may reflect the optimized DEX dosing (0.04 μg/kg/h) selected for postoperative intravenous analgesia.

### Inflammation

Previous studies have established anti-inflammatory properties of DEX,^25^ with CRP serving as an inflammatory marker, while acknowledging postoperative inflammation levels as potential confounders in assessing sleep quality effects of DEX.^21^ Meta-analytic evidence confirms the association of DEX with reduced CRP release^31^; however, this study observed insignificantly lower CRP levels on postoperative day 1 in group I than group P. A study on laparoscopic colorectal cancer surgery,^32^ in contrast to our findings, found that continuous intraoperative infusion of DEX was effective in suppressing inflammation and stress. However, it used, among others, tumor necrosis factor α, interleukin (IL)-8, and IL-6 as indicators of inflammation and stress.

**TABLE 6:** Univariable linear regression of sleep quality on postoperative day 1

Variable		β	P value	β (95% CI)
Group	I (reference)	–	–	–
	P	1.74	0.02	1.74 (0.35–3.14)
Sex	Men (reference)	–	–	–
	Women	–2.2	0.01	–2.2 (–3.74 to –0.67)
Age		0.02	0.56	0.02 (–0.06 to –0.11)
Dosage of remifentanil		0	0.33	0 (0–0)
Dosage of propofol		0	0.98	0 (0–0)
Dosage of norepinephrine		0	0.17	0 (0–0)
Dosage of rocuronium		0	0.89	0 (0.02–0.03)
C-reactive protein		0.03	0.17	0.03 (0–0.07)
White blood cell count		0.18	0.15	0.18 (–0.06 to 0.41)
Blood glucose		–0.13	0.31	–0.13 (–0.37 to 0.12)
Pain		0.03	0.86	0.03 (–0.26 to 0.31)

**TABLE 7:** Multivariable linear regression of sleep quality on postoperative day 1

Variable		β	P value	β (95% CI)
Group	I (reference)	–	–	–
	P	1.61	0.03	1.61 (0.22–3)
Sex	Men (reference)	–	–	–
	Women	–1.94	0.02	–1.94 (–3.52 to –0.35)
Age		0.02	0.61	0.02 (–0.06 to 0.1)
Dosage of remifentanil		0	0.93	0 (0–0)
Pain		0.08	0.54	0.08 (–0.18 to 0.34)

### Adverse effects

Compared with intraoperative DEX continuous infusion, DEX added to postoperative intravenous analgesia during laparoscopic gastrointestinal tumor resection surgery showed no significant differences in the incidence of intraoperative bradycardia, hypotension, or postoperative complications, including delirium, nausea, and vomiting, indicating comparable safety profiles of these 2 administration regimens.

Although no interaction was detected in the subgroup analyses, the point estimates for patient groups, such as chemotherapy and stoma populations, were still slightly lower than the overall mean. This suggests that future studies could expand the sample size of at-risk populations to confirm whether sleep interventions need to be adapted individually.

### Limitations

This study assessed the impact of different DEX administration strategies on postoperative day 1 sleep quality solely via the NRS, without sleep architecture monitoring (eg, polysomnography [PSG]), precluding direct comparison of their effects on sleep structure—a limitation necessitating future PSG studies to evaluate regimen-specific sleep architecture alterations.

Additionally, 3-day postoperative sleep quality follow-up may be insufficient to capture long-term effects, warranting extended retrospective analyses of DEX administration strategies.

The sample size calculation in this study was based on a parametric approach, but the actual data were analyzed using a nonparametric test, which may have slightly reduced the efficacy of the test. However, due to the large effect sizes observed and the fact that the shedding rate was taken into account, we believe that the results are still reliable. Future studies estimating sample size based on nonparametric assumptions or more conservative effect sizes are required.

## CONCLUSIONS

Addition of DEX to postoperative intravenous analgesia demonstrated superior efficacy in improving sleep quality on postoperative day 1 in gastrointestinal tumor surgery patients, as compared with intraoperative continuous DEX infusion, while achieving superior glycemic control at surgical conclusion.

## JOURNAL INFORMATION

Videosurgery and Other Miniinvasive Techniques is an official journal of the Videosurgery Foundation.
